# Deciphering the urinary microbiome and urological cancers: from correlation to mechanisms and treatment

**DOI:** 10.3389/fmicb.2025.1699308

**Published:** 2025-11-13

**Authors:** Zunwen Zheng, Deqian Xie, Yongzheng Han, Guandu Li, Shijin Wang, Xiaoman Zhang, Tao Huang, Wenfei Xu, Guangzhen Wu

**Affiliations:** 1 Department of Urology, The First Affiliated Hospital of Dalian Medical University, Dalian, Liaoning, China; 2 Zhejiang Key Laboratory of Multiomics and Molecular Enzymology, Yangtze Delta Region Institute of Tsinghua University, Zhejiang, Jiaxing, China

**Keywords:** urinary microbiome, urological cancer, microbial ecology, gut–prostate axis, microbial biomarker, immune modulation

## Abstract

Given that the sterility of urine and the urinary tract has been questioned by research, urinary microbiome dysbiosis has been recognized as one of the potential cancer-promoting factors. The composition of the urinary microbial community in healthy individuals has a relatively high similarity at the phylum level, with factors like age and gender influencing the expression and distribution. In contrast, the urinary microbiome of patients with urologic cancers shows significant variability and diversity depending on the type of cancer. Most of the early studies focused on the distribution, aggregation, and expression of microbiota in urologic cancers, warranting advanced studies on the causal relationship between microbes and urologic cancers. Bladder and prostate cancer tumorigenesis and progression can be influenced by microbes through chronic inflammatory or immunomodulatory pathways making them cancer models strongly associated with the urinary microbiome. Here, we summarize the expression characteristics of the microbiomes associated with these cancers and analyze the pathophysiological mechanisms and signaling pathways of the microbiome in the tumor promotion or suppression. By examining the role played by the urinary microbiome in the pathogenesis of urologic cancers, we assess the potential of specific microbial groups as biomarkers for diagnosis and surveillance. Additionally, involving the microbiome or using adjunctive participation in tumor therapy is becoming an emerging cancer treatment option. Improving urinary microbial homeostasis in urinary cancers by direct treatment with microbial products, microbial co-immunotherapy, probiotic-assisted therapy, and fecal microbial transplantation may broaden the scope of therapy and enhance the efficacy of conventional medicines.

## Highlights

- The urinary microbiota in healthy individuals is influenced by gender, age, and hormones.
- The urinary microbiome can serve as biomarkers for diagnosis and prognosis of urological tumors.
- Intestinal microbiome and its metabolites have a regulatory role in urological cancers.
- Microbiome-assisted antitumor therapies represent a novel strategy for treating urological cancers.

## 1 Introduction

Urological cancers are malignant tumors arising from organs of the urinary tract, primarily including renal cancer, bladder cancer, urothelial carcinoma, and prostate cancer. In clinical practice, malignant tumours of the urinary system, such as muscle-invasive bladder cancer and advanced prostate cancer, exhibit substantial invasiveness and a high risk of metastasis. This often results in a significant deterioration in patients’ quality of life and prognosis. Urinary system cancers currently have increasing incidence and mortality rates globally, posing a serious threat to human health worldwide (Dy et al., 2017; Li C. et al., 2022).

According to the 2022 World Health Organization’s International Agency for Research on Cancer (IARC) statistics, prostate cancer and bladder cancer accounted for 7.3 and 3.1% of newly diagnosed global cancer cases, ranking 4th and 10th, respectively. In terms of cancer-associated mortality, prostate cancer ranks 8th, accounting for 4.1% of all cancer deaths worldwide. Epidemiological statistics confirm that urological tumors are a significant global disease burden, as evidenced by significantly higher morbidity and specific mortality patterns. These statistics continue to threaten the quality of survival and clinical prognosis of patients (Bray et al., 2024). Over the past decade, the field of urologic oncology has undergone a transformative evolution, driven by paradigm shifts in tumor pathophysiology, innovative diagnostic biomarker discovery, and treatment optimization, which have resulted in significant improvements in clinical outcomes and survival trajectories (Dy et al., 2017; Moch et al., 2022; Netto et al., 2022).

Urinary tract cancers arise from multifactorial interactions between environmental determinants, genetic factors, and host susceptibility. The most prevalent contributors are occupational exposure to carcinogens, persistent inflammatory stimulation and germline genomic abnormalities (Volanis et al., 2010; Sonkin et al., 2024). Microbiome ecology is the field dedicated to investigating the interrelationships between microorganisms, their habitats, and symbionts. It encompasses research into the dynamic distribution, compositional hierarchy, and functional networks of microbes within ecosystems (Godoy-Vitorino, 2019). This has fundamentally transformed our understanding of the mechanisms underlying tumourigenesis within the urinary tract of the host. As the factor most closely linked to the host-environment interface, urinary tract microbiota influence tumour initiation, progression and therapeutic response. Disruption to homeostatic balance caused by ecological imbalance is regarded as a potential carcinogenic promoter (Bučević Popović et al., 2018; Bi et al., 2019).

Cutting-edge research on urological tumours has prioritised models of carcinogenesis driven by the microbiome. This undoubtedly represents the latest paradigm shift in oncological research. Pioneering studies have elucidated how host-associated urological microbiota can regulate malignant transformation through pathways including chronic inflammatory cascades, virulence factor secretion, immune checkpoint modulation and genomic instability (Sepich-Poore et al., 2021; Liu et al., 2024b). Current tumour therapies now extend beyond the traditional triad of surgery, chemotherapy and radiotherapy, incorporating emerging approaches such as molecularly targeted agents, antibody-drug conjugates and immune checkpoint inhibitors (Sonkin et al., 2024; Li J. et al., 2025). Investigating the role of the microbiome within these therapeutic approaches is highly promising. A deeper understanding of the relationship between the urological microbiome and cancer could enhance our knowledge of carcinogenic mechanisms, providing novel insights and strategies for preventing, diagnosing, and treating urological cancers.

## 2 Urinary tract microbiome in healthy individuals: diversity and influencing factors

### 2.1 Composition of the major microbiome of the urinary tract in healthy individuals

For a long time, urine has been considered sterile due to its low pH, high urea concentrations, and other factors unfavorable to microbial growth (Colella et al., 2023; Ipe et al., 2016). However, analysis of the urinary microbiome with developing microbiome technologies, high-throughput sequencing techniques, such as 16S rRNA sequencing, macro-genomics, and single-cell sequencing revealed that there are diverse but low-biomass microbial communities in the urinary tract of healthy individuals (Thomas-White et al., 2018). Emerging evidence suggests that the urinary microbial community in healthy individuals is not static but dynamically regulated by multiple host and environmental factors, including immune status, hormonal levels, dietary habits, and aging (Russo et al., 2024). Notably, gender is one of the primary determinants influencing the composition of the urinary microbiome, which will be discussed in greater detail in the following section.

A diverse microbial community inhabits the healthy urinary tract. At the phylum level, *Firmicutes, Actinobacteriota (Actinobacteria)*, *Proteobacteria*, and *Bacteroidota* are most frequently detected; among fungi, *Ascomycota* is commonly reported. At lower taxonomic ranks, representative genera include *Lactobacillus* (family Lactobacillaceae), coagulase-negative *staphylococci* (CoNS; genus *Staphylococcus*, family Staphylococcaceae), *Corynebacterium* (family Corynebacteriaceae), *Neisseria* (family Neisseriaceae), *Arthrobacter* (family Micrococcaceae), and several obligate anaerobes (e.g., *Finegoldia, Anaerococcus,* and *Peptoniphilus*) (Neugent et al., 2020; Aragón et al., 2018). Additionally, recent studies on the urinary tract mycobiome in healthy individuals have identified many fungal communities, including *Dothideomycetes*, *Saccharomycetes*, *Exobasidiomycetes, Microbotryomycetes*, and *Candida*, which can be detected in the urine of some individuals (Neugent et al., 2020). Although the abundance of these fungi is low, their presence suggests that the urinary microbiota is more complex than previously understood. Recent studies have confirmed that relevant fungal components (mycobiome) activate the IL-1β/IL-6/IL-23–Th17 axis via receptors such as Dectin-1/TLR2/4, thereby shaping a pro-tumour inflammatory microenvironment (Xu et al., 2025; Saftien et al., 2023). Similarly, some studies suggest that fungi and bacteria exhibit co-increases or decreases within gastrointestinal tumours. This alters the epithelial barrier and antigen presentation, subsequently affecting immune cell infiltration and function (Li et al., 2024). These fungi may interact with resident bacterial populations through competitive or synergistic mechanisms, potentially influencing microbial equilibrium, mucosal immunity, and even host susceptibility to urinary tract disorders.

### 2.2 Main factors influencing urinary tract microorganisms in healthy individuals

Gender is a significant factor influencing the urinary tract microbiome in healthy individuals. Studies have demonstrated that higher estrogen levels in women maintain the acidic environment of the urinary tract by promoting lactic acid production through *Lactobacillus* in their urinary flora, which inhibits pathogen colonization to a certain extent (Shoemaker and Kim, 2021). As a result, women generally exhibit a *Lactobacillus*-dominant urinary microflora, including *Lactobacillus*, *Prevotella*, *Gardnerella*, *Peptoniphilus*, and *Dialister*, of which *Lactobacillus* is dominant (Colella et al., 2023). Unlike the *Lactobacillus*-dominated flora in women, the male urinary microbiota is dominated by *Corynebacterium* (Roth et al., 2023). This difference may be due to anatomical differences: the male urethra is longer, which may reduce the ability of certain bacteria to colonize the urethra, influencing the microbial composition (Hourigan et al., 2020). Interestingly, the relative abundance of several genera such as *Prevotella, Escherichia, Enterococcus, Streptococcus*, and *Citrobacter* showed no significant sex-related differences, suggesting that gender does not impact all microbial taxa (Fouts et al., 2012) (Table 1).

**Table 1:** Healthy male and female urinary microbiota.

Category	Microbiota composition	References
Male urinary dominant microbiota	*Lactobacillus*, *Streptococcus*, Sneathia, Mycoplasma, Ureaplasma, *Corynebacterium*	Roth et al. (2023), Nelson et al. (2012), Lewis et al. (2013)
Female urinary dominant microbiota	*Lactobacillus*, *Prevotella*, *Acinetobacter*, *Gardnerella*, *Peptoniphilus*, *Staphylococcus*, *Psychrobacter*, *Dialister*	Lewis et al. (2013), Siddiqui et al. (2011)
Gender-neutral microbiota	*Prevotella*, *Escherichia*, *Enterococcus*, *Streptococcus*, *Citrobacter*	Aragón et al. (2018), Pederzoli et al. (2020)

Recent studies have demonstrated that the urinary microbiota undergoes dynamic compositional changes at various life stages, regulated by age-related physiological shifts. Notably, the urogenital microbiota of the older age group (60–70 years) shows an enrichment of some under-characterized genera, such as *Jonquetella* spp., *Parvimonas* spp., *Proteiniphilum* spp., and *Saccharofermentans* spp. Despite the fact that these microorganisms exhibit dependent colonisation in the elderly population, further research is required to elucidate their metabolic functions and physiological roles in a healthy urinary system. Elucidating the functions of these poorly characterised bacterial genera will not only fill gaps in urinary microbiome research in older adults, but also provide new insights into the pathogenesis of urinary tract diseases in this age group (Colella et al., 2023; Roth et al., 2023; Flores-Mireles et al., 2015).

## 3 Microbiome and urologic malignancies

**Figure 1:**
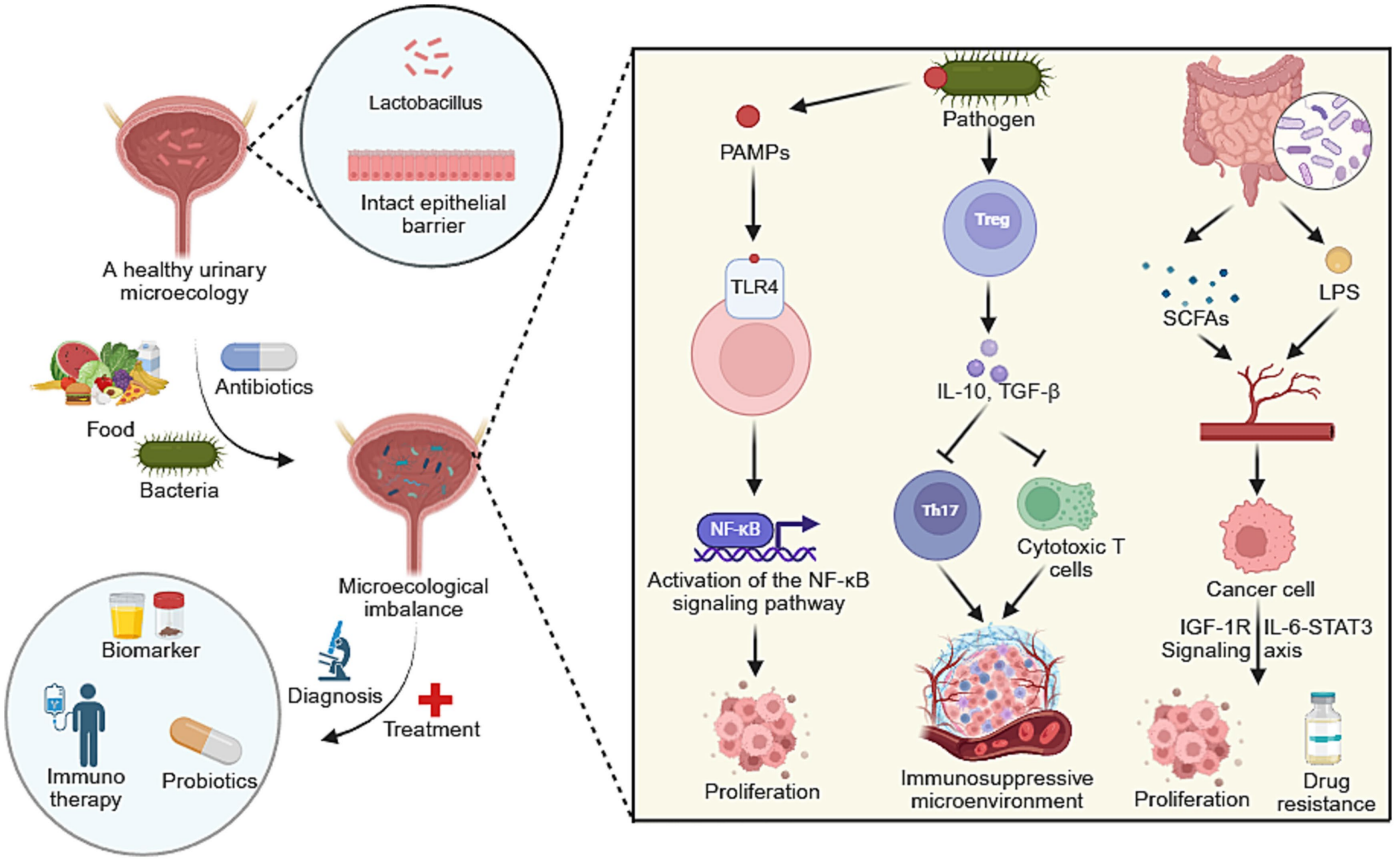
A conceptual figure illustrating the role of the urinary microbiome in urological cancers. Microecological dysbiosis drives tumor initiation and progression through key mechanisms including activation of pro-inflammatory signaling pathways and the establishment of an immunosuppressive microenvironment. This mechanistic understanding links microbial dysbiosis directly to novel diagnostic biomarkers and emerging therapeutic strategies, such as immunotherapy and microbiome modulation. Created with BioRender.com.

With the gradual advancement and improvement of high-throughput sequencing technology and multi-omics approaches, the research on urinary microbes and urological tumors mainly focuses on three aspects:

Studying the association between the urinary microbiome and urological tumors: urinary pathogens induce chronic inflammation through specific signaling pathways and their bacterial metabolites, resulting in the tumor regulation of host–microbe interactions (Xu et al., 2014).
Exploring the urinary microbiome as a potential biomarker for urinary tract tumors to improve early diagnosis (Wu et al., 2024).
Probing promising microbial therapies for urinary tract tumors. Although growing evidence progressively outlines the role of microbes in urologic oncology, the mechanisms of the relationships between the urinary tract microbiome and tumor transformation are yet to be understood.
This review focuses on two central urologic malignancies, bladder and prostate cancer, systematically aiming to resolve their underlying microbial etiopathogenesis and integrate modern research pathways from microbial dysbiosis patterns to potential oncogenic factors (Figure 1).

### 3.1 Bladder cancer

#### 3.1.1 Bladder microbiome characteristics in bladder cancer patients: commonalities and differences

Early studies were constrained by traditional microbiological methods and struggled to accurately determine the true microbial composition of the urinary tract. However, research based on 16S rRNA sequencing has confirmed that the bladder is not sterile, but rather occupies a niche with a low microbial load (Stamatakos et al., 2024). In comparison to the extremely high diversity and active physiological functions of gut microbiota, bladder microbiota exhibit relatively low density. Its primary function is to maintain the homeostatic balance of the urinary system. This implies that sequencing results are more susceptible to interference from exogenous microorganisms. Multiple existing studies demonstrate that imbalances in the urinary microbiome of bladder cancer patients occur at the community level rather than being confined to a single pathogenic microorganism. Compared to healthy controls, bladder cancer-associated samples consistently exhibit elevated total bacterial abundance and systematic reshaping of community structure. This is reflected in significant alterations in both *α* diversity (species richness/evenness) and *β* diversity (inter-community variation) (Pederzoli et al., 2020; Wu et al., 2018b). This imbalance signifies a shift in the urinary microbial ecosystem as a whole, moving from a state of homeostasis towards inflammation rather than being dominated by a single constant carcinogenic bacterium (Tables 2, 3).

**Table 2:** Methods and key findings on the urinary microbiome and bladder cancer.

Sample type	Research methods	Key findings	Year	References
Clean midstream urine	16S rRNA sequencing	Differentiated healthy urine microbiome from asymptomatic bacteriuria by taxonomic and proteomic profiles.	2012	Fouts et al. (2012)
Catheter-collected urine from bladder cancer patients	16S rRNA sequencing	Urinary oxygen tension correlated with microbiome composition (correlation/regression models).	2019	Shannon et al. (2019)
Tissue samples collected from cancerous bladder mucosa and adjacent normal tissues	16S rRNA sequencing and PCR amplification of the V3-V4 region of the 16S rRNA gene	*Proteobacteria* predominant; species richness and Shannon index decreased in tumor (Kruskal–Wallis).	2019	Liu et al. (2019)
Catheter-collected urine samples from male bladder cancer patients	16S rRNA gene sequencing	*Veillonella* and Corynebacterium increased, *Ruminococcus* decreased; *Burkholderiaceae* elevated in bladder lavage; midstream urine enriched in *Streptococcus, Enterococcus, Corynebacterium*, and *Fusobacterium* (*p* values reported).	2021	Oresta et al. (2021)
Urine and tumor tissue from patients who underwent radical cystectomy, and normal urine and bladder tissue from healthy controls	16S rRNA gene sequencing and analyses of α-diversity & *β*-diversity	Higher bacterial load was observed in bladder cancer with significant α/β-diversity changes. Urine–tissue taxa overlapped by about 81% in men and up to 98% in women, while tumor tissue was enriched for Betaproteobacteria, Burkholderiales, and Burkholderia.	2020	Pederzoli et al. (2020)
Midstream urine from bladder cancer patients at different stages and non-cancerous control patients	16S rRNA gene sequencing and α-diversity & β-diversity analysis	Higher abundance of *Acinetobacter* and other genera in bladder cancer; some results lacked statistical specification.	2018	Wu et al. (2018a)
Urine samples from bladder cancer patients	16S rRNA gene sequencing	Bladder cancer samples showed an abundance of four common core bacterial taxa, *Acinetobacter, Rubrobacter, Geobacillus*, and *Rhizobiales* than in non-cancerous samples.	2019	Mai et al. (2019)
Consecutive urine samples from bladder cancer patients and healthy volunteers.	16S rRNA gene sequencing	Greater bacterial abundance associated with higher recurrence/progression risk strata; tumor grade showed no clear association with microbiome features.	2021	Hussein et al. (2021)
Urine samples from bladder cancer patients	Amplicon-based next-generation sequencing	*Actinomyces* and *Actinobacteria* were more abundant in bladder cancer. *Firmicutes*, *Actinobacteria*, *Proteobacteria*, and *Bacteroidetes* together accounted for approximately 94.4% of the detected taxa (*p* < 0.05).	2019	Bi et al. (2019)

**Table 3:** Biological and clinical implications distilled from the same studies.

Study (Year)	Statistical significance	Biological relevance	Clinical importance
Fouts et al. (2012)	Yes	Microbiome–metaproteome differences distinguish healthy vs. asymptomatic bacteriuria.	Encourages phenotype-integrated microbiome and proteome approaches.
Shannon et al. (2019)	Yes (correlation/regression models).	Urinary oxygen tension as environmental selector shaping communities.	Motivates integrated environment–microbiome–tumor studies.
Liu et al. (2019)	Yes (Kruskal–Wallis; multiple-testing handling variably reported).	Tissue-level dysbiosis with *Proteobacteria* predominance.	Suggests integrating tissue microbiome into pathologic assessment (exploratory).
Oresta et al. (2021)	Yes (multiple genus-level differences; *p*-values reported; FDR inconsistent).	Stage-associated genus signatures; sample type critically shapes signals.	Hypothesis-generating for staging/subtyping; requires external validation.
Pederzoli et al. (2020)	Yes (α/β-diversity and bacterial load).	Gram-negative/LPS-linked ecological shift consistent with inflammation, barrier disruption, immune modulation.	Under rigorous sampling, urine can partially proxy tissue ecology; supports non-invasive risk stratification (exploratory).
Wu et al. (2018a)	Partial/NR.	Acinetobacter and related genera enriched—signals require verification.	Candidate taxa need stronger statistics and external validation.
Mai et al. (2019)	Yes (cross-dataset synthesis).	Methodological contribution: Identified candidate bacterial taxa consistently detected across independent datasets, providing a reference framework for biomarker discovery.	Useful methodological reference; requires prospective validation.
Hussein et al. (2021)	Yes (greater bacterial abundance was associated with higher recurrence and progression risk strata).	Microbiome likely influences disease trajectory more than initial grade.	Source for exploratory prognostic features; needs prospective validation.
Bi et al. (2019)	Yes (*p* < 0.05).	*Actinobacteria*/*Actinomyces* shifts align with ecological differences.	Conceptual basis for biomarker/therapeutic targeting (validation needed).

At the taxonomic level, the enrichment of *Acinetobacteraceae*/*Acinetobacter* and *Sphingobacteriaceae/Sphingobacterium* has been repeatedly observed across multiple studies (Wu et al., 2018b). These are predominantly opportunistic Gram-negative pathogens. The enrichment of these bacteria, which carry pathogen-associated molecular patterns such as lipopolysaccharides, suggests upregulation of inflammation-associated ecological pathways (e.g., LPS-triggered mucosal immune activation, epithelial barrier stress, and altered metabolic pathways). This represents a functional shift. Furthermore, different cohort studies report relatively large proportions of the *Actinobacteria* and *Anaplasma* phyla constituting residual or non-dominant communities within these samples (Hussein et al., 2021). This underscores the need for particular caution when estimating relative microbial abundance, accounting for the influence of population background, sampling methods, sequencing protocols, and decontamination strategies on experimental outcomes.

Where do these differences originate? The most commonly used samples for bladder cancer microbiome studies are midstream clean-catch urine and catheterised urine from bladder cancer patients. Clean-catch midstream urine is more susceptible to contamination from urethral and external genital flora, whereas catheterised urine reduces upstream contamination, more closely approximates the bladder microenvironment, and significantly diminishes the influence of urethral flora, thereby more accurately reflecting the microbial composition of the bladder microenvironment (Hourigan et al., 2020; Bajic et al., 2020). Building on this, a recent study in European Urology Open Science showed that first-morning urine yields urobiome profiles broadly comparable to catheterised urine in patients undergoing evaluation for bladder cancer. Compared with catheterised specimens, first-morning urine (FMU) offers multiple advantages: it is non-invasive, lower cost, and readily scalable, making it better suited for large, longitudinal cohorts. FMU also improves patient acceptability and avoids procedure-related iatrogenic risks. Accordingly, when paired with rigorous negative controls and decontamination procedures, FMU can serve as a feasible, non-invasive alternative specimen, enabling larger prospective studies and improving research accessibility (Nardelli et al., 2024).

In investigations of dysbiosis, some research has focused on exploring the fungal microbiome in the urine of bladder cancer patients (Wu et al., 2018b; Mercier et al., 2023). Notably, several studies have detected fungal signals in bladder cancer-associated samples, such as the presence of the phylum *Ascomycota* (Mercier et al., 2023; Ackerman and Underhill, 2017). This corroborates earlier findings indicating that the urinary fungal microbiome exhibits high diversity and significant inter-individual variation. This suggests the bladder may be regarded as a low-biomass cross-domain (bacterial-fungal) microecosystem; within this system, minute alterations in its components can influence community functional states, subsequently affecting the host’s inflammatory response. In other disease systems, alterations in fungal microbiota are frequently associated with major pathologies in immunocompetent hosts. Although precise mechanisms remain unclear, these findings suggest the role of fungi in tumour microenvironment development may be underestimated. Larger-scale studies are required to further elucidate the function of fungal microbiota in bladder cancer (Ackerman and Underhill, 2017).

#### 3.1.2 Factors influencing differences in the urinary microbiome and prognostic associations in patients with bladder cancer

A recent case–control study, which was stratified by gender, found that bladder cancer samples which had not received neoadjuvant therapy exhibited higher total bacterial biomass than healthy controls. This was accompanied by significant alterations in community diversity, with shifts in both *α*- and *β*-diversity. This pattern of dysbiosis proved statistically significant in both male and female subgroups, whereas differences in bladder tissue samples were negligible. There was high community composition overlap between urine and tissue microbiomes: approximately 81% of taxa were shared between sample types in male specimens, rising to 98% in female samples (Pederzoli et al., 2020). These findings suggest that urine may serve as a surrogate window into local tissue ecology to some extent, provided stringent sampling and quality control protocols are followed. Tumour tissues exhibited significant enrichment of the *β*-*Proteobacteria* class, Burkholderiales order and Burkholderia genus, a pattern consistent with mucosal inflammatory activation pathways, compromised epithelial barrier function and altered immune regulation (Pederzoli et al., 2020).

Another study demonstrated that patients with higher bacterial abundance were more likely to be classified as high risk for recurrence/progression (Wu et al., 2024). However, no consistent association was found between tumour histological grade and microbial characteristics, suggesting that the microbiome influences disease progression (recurrence/progression) rather than determining initial malignancy (Hussein et al., 2021). Similarly, another study found a negative correlation between the copy number of the Ureaplasma strain CAG:581 in urine and patient survival rates (Zhang Y. et al., 2023). Furthermore, CAG:581 could potentially serve as a prognostic biomarker for monitoring non-muscle-invasive bladder cancer (NMIBC). These findings on differences in the bladder cancer microbiome lay the groundwork for future exploration of its potential as a non-invasive biomarker for predicting recurrence risk and disease progression.

#### 3.1.3 Potential microbial carcinogenesis in bladder cancer

Chronic inflammation is a common microbial cancer-promoting pathway in tumorigenesis, and bladder cancer is no exception. Alterations in the urinary microbiota trigger chronic inflammation, disrupting urinary tract homeostasis and leading to the development of highly prevalent urological disorders such as interstitial cystitis and urgency urinary incontinence (Fouts et al., 2012; Pearce et al., 2014). Chronic infection with uropathogenic bacteria, such as *Staphylococcus aureus*, *Escherichia coli*, and *Fusobacterium*, induces chronic tissue damage accompanied by a corresponding increase in persistent cell renewal and restorative hyperplasia. During this process, n -n-nitrosamine compounds, which are capable of accelerating the oncogenic process by causing DNA alkylation and p53 gene mutations, can be detected in the patient’s urine, with studies documenting that such bacteria can mediate nitrosative reactions (Sanli et al., 2017; McLellan and Hunstad, 2016; Mostafa et al., 1999). The persistence of the inflammatory response is regulated by mechanisms of pathogen-associated molecular patterns (PAMPs) of microbial origin. These evolutionarily conserved molecular motifs are recognized specifically by germline-encoded pattern recognition receptors (PRRs), triggering a Toll-like receptor (TLR) cascade response, leading to oncogenic signaling network activation, including Janus kinase (JAK)-signal transducer and activator of transcription 3 (STAT3) transcriptional reprogramming, Nuclear factor kappa B (NF-κB)-driven metabolic adaptations, and phosphoinositide 3 kinase (PI3K)/Akt/mammalian (or mechanistic) target of rapamycin (mTOR)-mediated cell survival pathways. Notably, in bladder-like organ invasion experiments using uropathogenic *E. coli* clinical isolates showed NF-κB activation, accompanied by the activation of anti-apoptotic signaling and the overproduction of interleukin (IL)-1β/IL-6, which in turn inhibited apoptosis while increasing inflammatory responses to fuel tumorigenesis (El-Mosalamy et al., 2012; Heidar et al., 2023).

Current research on the bladder cancer microbiome remains limited. Most studies are still in the ‘descriptive’ phase. Due to differences in sample collection sites (midstream urine samples, catheterised urine samples, bladder tumor samples), the results lack consistency and comparability. There are also areas for improvement in study design. Incorporating long-term follow-up of patients could further validate the study results. Additionally, validating existing findings in animal models is essential. Addressing these issues is a necessary step for bladder cancer microbiome research to advance to the next phase (Yacouba et al., 2022; Mirzaei et al., 2021; Parra-Grande et al., 2021).

### 3.2 Prostate cancer

#### 3.2.1 Microbiota alterations in prostate cancer patients

Recent studies indicate that the urinary tract microenvironment in male patients with prostate cancer exhibits abnormal alterations, affecting both prostate tissue biopsy specimens and the urinary microbiome (Sfanos et al., 2018). Multiple cohort studies have identified significantly higher abundances of bacterial genera, such as *Cutibacterium* spp., *Streptococcus* spp., and *Escherichia coli*, in prostate tissue or urine samples from prostate cancer patients than in control groups (Davidsson et al., 2016; Liss et al., 2018; Jain et al., 2020). These microorganisms have been shown to be linked to chronic inflammation and immune regulation within the tumour microenvironment. This supports the idea that localised microbiota dysbiosis contributes to the development and progression of prostate cancer.

Concurrently, analyses of the gut microbiome—primarily conducted via faecal sequencing—revealed that patients with prostate cancer (PCa) exhibit reduced microbial diversity in the gut and pronounced taxonomic alterations compared to healthy individuals. These systemic changes may have an impact on other parts of the body through metabolic and immune pathways, such as the short-chain fatty acid/G protein-coupled receptor/insulin-like growth factor 1 (SCFA/GPCR/IGF-1) pathway and the lipopolysaccharide-interleukin 6/signal transducer and activator of transcription 3 (LPS–IL-6/STAT3) pathway. These findings support the ‘gut-prostate axis’ theory, which suggests that gut dysbiosis can affect the biological characteristics of prostate tumours by releasing circulating metabolites and immune signals.

These data reveal two levels of dysbiosis associated with prostate cancer (PCa): (1) localised alterations in the prostate/urinary tract microbiota linked to inflammatory remodelling of the tumour microenvironment, and (2) systemic alterations in the gut microbiota which may regulate prostate biology through endocrine, metabolic and immune pathways. Distinguishing between these two microbial communities provides crucial insights into the role of the microbiota in prostate cancer and lays the groundwork for the development of microbiome-based diagnostic and therapeutic strategies (Fujita et al., 2023; Golombos et al., 2018) (Table 4).

**Table 4:** Microbial shifts in the prostate cancer microenvironment.

Microbial characteristics	Prostate cancer (PCa) patients	Controls	References
Abundance			
Propionibacterium	Significantly increased	Lower levels	Davidsson et al. (2016)
*Streptococcus*	Increased	Lower levels	Liss et al. (2018)
*Escherichia*	Certain strains increased	Normal levels	de Bono et al. (2020)
Short-chain fatty acids (SCFAs)	Increased	Normal levels	Matsushita et al. (2021a)
Diversity			
Gut microbiota	Decreasing trend	Higher diversity	Fujita et al. (2023), Yadav et al. (2024)

#### 3.2.2 Key microbiome in prostate cancer

##### 3.2.2.1 Cutibacterium acnes

*Cutibacterium acnes* is one of the typical flora in the prostate microenvironment. For instance, Davidsson et al. showed that *Cutibacterium* *acnes* is more common in men with PCa than those without PCa (Davidsson et al., 2016). *Cutibacterium acnes* induces a chronic inflammatory response (Davidsson et al., 2016; Shinohara et al., 2013; Kistowska et al., 2015), and chronic inflammation may contribute to PCa development and progression through several mechanisms (de Bono et al., 2020). In addition, *Cutibacterium acnes* can regulate the local immune microenvironment, and by inducing regulatory T-cell (Treg) infiltration and inhibiting the function of Th17 cells, leading to an immunosuppressive state in the tumor microenvironment of PCa patients and inhibit the body’s immune surveillance and elimination of tumor cells, thus promoting tumor growth and metastasis (Radej et al., 2022).

##### 3.2.2.2 Streptococcus

A report highlighted differences in the composition of the gut microbiome (GM) between PCa patients and non-PCa individuals, showing a decrease in the diversity of GM in PCa patients compared to controls (Huang et al., 2024). A related study found a significant increase in the relative abundance of streptococci in PCa patients by sequencing rectal swabs, suggesting that streptococci may be associated with PCa development (Liss et al., 2018). Further, streptococci may contribute to the development and progression of PCa through chronic inflammation-induced tissue damage, angiogenesis, and tissue repair (De Marzo et al., 2007; Nakai and Nonomura, 2013). Streptococci can interact with other microorganisms (e.g., Bacteroides) to influence the metabolic functions of the gut microbiome and indirectly impact PCa development and progression (Liss et al., 2018).

##### 3.2.2.3 *Escherichia* (*Escherichia coli*)

*Escherichia coli* has been detected by researchers in both malignant and benign prostate tissues. However, as clinicians typically perform prostate biopsies through the rectum, the procedure may inadvertently introduce rectal *E. coli* into prostate specimens. This contamination complicates the interpretation of results, as no definitive causal relationship between *E. coli* and prostate pathology has been established currently (Jain et al., 2020). To address this issue, some studies have adopted transperineal biopsy techniques, which bypass the rectal mucosa, reducing the risk of microbial contamination (Wagenlehner et al., 2014). A subsequent study also confirmed the feasibility of this method. Chen et al. similarly focused on transperineal prostate microbiome biopsy. In this experiment, MRI-guided sampling was used to ensure accuracy. This also significantly reduced faecal microbiome interference, enabling us to more accurately assess the potential role of *Escherichia coli* in the development of prostate cancer (Chen et al., 2024).

Previous studies suggest that *E. coli* may promote malignant transformation of prostate epithelial cells by inducing chronic inflammation in prostate tissues, which in turn activates a cascade of pathological responses. These include abnormal cell proliferation, pathologic angiogenesis, and dysregulation of tissue repair mechanisms, a series of processes that can promote the malignant transformation of prostate epithelial cells and continue to drive PCa progression (de Bono et al., 2020). Additionally, *E. coli* may also act in concert with other pathogens, such as *Cutibacterium acnes* and *Neisseria gonorrhoeae*, to cause long-term chronic inflammation that promotes the development of PCa (de Bono et al., 2020).

#### 3.2.3 Influence of the microbiome on the “gut-prostate axis” hypothesis

The human gut microbiota is a dense, metabolically active ecosystem consisting of bacteria, archaea, viruses and fungi (Sun et al., 2025). This ecosystem influences host physiology by fermenting dietary substrates (e.g., producing short-chain fatty acids), training mucosal immunity and maintaining epithelial barrier integrity. Dysbiosis can reprogramme systemic immunity and metabolism via microbial metabolites and pathogen-associated molecular patterns. This has downstream effects on distant organs, including the prostate, through circulatory and neuroendocrine pathways (Sun et al., 2023).

The “gut-prostate axis” is a hypothesis that uses the gut microbiome as a connecting bridge to explore the relationship between the gut and the prostate. The hypothesis suggests that the gut microbiome influences prostate health through metabolites and immunomodulatory mechanisms (Matsushita et al., 2023). Dysbiosis of the gut flora may lead to impaired intestinal barrier function, allowing bacteria and their metabolites to enter the circulation and affecting the prostate microenvironment. To systematically elucidate the gut–prostate axis, the following sections shall provide detailed descriptions through three complementary pathways: metabolic pathways (SCFAs, GPCRs, and IGF-1), immune pathways (LPS, IL-6/STAT3), and endocrine pathways (microbiome modulates androgens and AR signaling).

Gut microbiota ferment dietary fibre to produce short-chain fatty acids (SCFAs), primarily comprising acetate, propionate, butyrate and isobutyrate (Koh et al., 2016). These small molecules enter the liver via the portal vein and subsequently the systemic circulation, where they are taken up by peripheral tissues through monocarboxylate transporters, such as MCT1. At target cell surfaces, they act as ligands to activate multiple G protein–coupled receptors (GPCRs), notably FFAR2 (GPR43), FFAR3 (GPR41), and HCAR2 (GPR109A) (Li S. et al., 2025). Within the prostate microenvironment, SCFAs trigger downstream signalling through these GPCRs, subsequently cross-talking with the PI3K–AKT–mTOR and MAPK/ERK pathways, while upregulating insulin-like growth factor-1 (IGF-1)-related signalling axes (Li S. et al., 2025). IGF-1, a pivotal growth factor regulating cell proliferation, differentiation, and survival, is secreted systemically by organs such as the liver, whilst also being locally induced within the microenvironment. Activation of its receptor promotes prostate cell cycle progression, anti-apoptosis, and metabolic reprogramming, often accompanied by upregulation of oncogene expression (Matsushita et al., 2021a). Consequently, the pathway “SCFA→GPCR→IGF-1” constitutes a viable metabolic route from intestinal lumen metabolites to prostatogenic signals.

**Figure 2:**
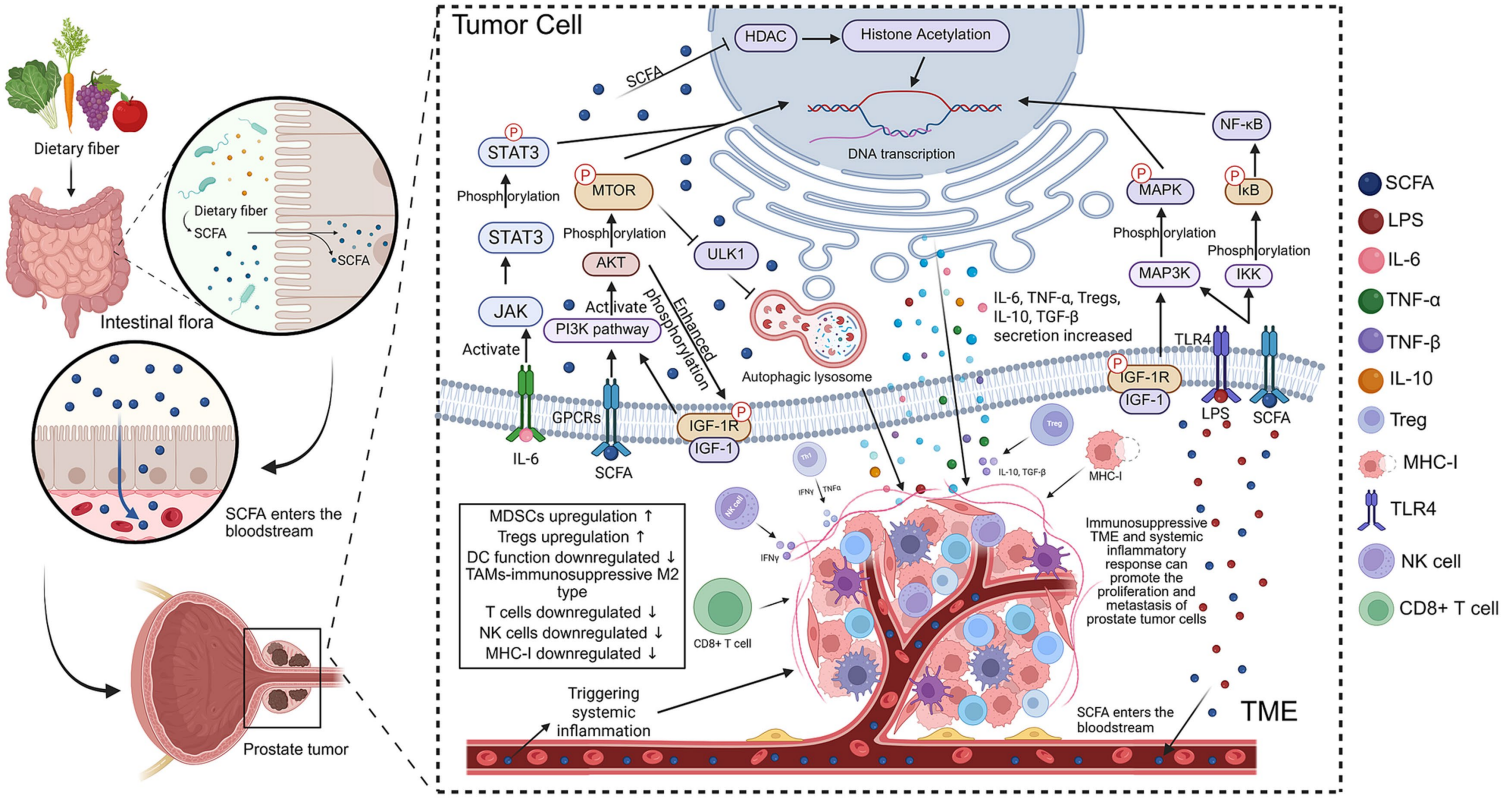
The figure illustrates the role of short-chain fatty acids (SCFAs) in the prostate-gut axis and their carcinogenic mechanisms in prostate cancer cells. It explains how SCFAs are produced from dietary fibers, metabolized by gut microbiota, enter the bloodstream, and influence the immune environment of prostate tumors. (1) SCFA production and entry into the bloodstream: Dietary fibers (such as vegetables, fruits, etc.) are metabolized in the gut to produce SCFAs, which enter the bloodstream through the intestinal wall and circulate throughout the body. (2) Regulation of gut immune responses: SCFAs activate immune cells like regulatory T-cells (Treg) and promote the secretion of pro-inflammatory factors and cytokines such as interluekin (IL)-6 and tumor necrosis factor-alpha (TNF-*α*). These cytokines further influence the immune cells in the tumor microenvironment (TME), including tumor-associated macrophages (TAMs) and dendritic cells (DCs), leading to an immune-suppressive response. (3) Immune escape mechanisms of prostate tumor cells: SCFAs activate multiple signaling pathways, including mitogen-activated protein kinase (MAPK) and phosphoinositide 3-kinase (PI3K) pathways, by stimulating receptors on the surface of prostate cancer cells like toll-like receptor 4 (TLR4) and IGF-1R, enhancing cancer proliferation and metastasis. SCFAs also induce histone acetylation and inhibit histone deacetylase (HDAC), altering gene expression in tumor cells. (4) SCFAs and systemic inflammatory response: SCFAs promote systemic inflammation, which further exacerbates the immune suppression within the TME by upregulating myeloid-derived suppressor cells (MDSCs), reducing T cell and NK cell function, and decreasing MHC-I expression. The outcome is promoting the proliferation and metastasis of prostate cancer cells (Created with BioRender.com).

In populations at high risk of prostate cancer, gut microbiota dysbiosis, which is associated with dietary and smoking factors, often manifests as reduced diversity and altered community composition. This is characterised by relative increases in SCFA-producing strains, such as Lachnospira, Rikenellaceae and Alistipes (Fujita et al., 2023; Pernigoni et al., 2023; Matsushita et al., 2021b). While deprivation of gut-derived SCFAs inhibits PCa growth *in vivo* (Matsushita et al., 2021a), the imbalance itself initiates a more direct pro-tumour pathway by increasing intestinal barrier permeability. LPS from Gram-negative bacteria enter the bloodstream via the portal vein, inducing mild endotoxinaemia. This LPS is recognised by TLR4 on epithelial/mesenchymal cells and myeloid immune cells within the prostate microenvironment, triggering a MyD88/TRIF-dependent NF-κB emergency response and IL-6 release (Matsushita et al., 2022; Zhong et al., 2022). The secreted IL-6 subsequently activates the JAK–STAT3 cascade in tumour and immune cells, driving a series of tumour progression-associated phenotypes, including the upregulation of proliferation and anti-apoptotic programmes (e.g., target genes such as BCL-XL and Survivin), the maintenance of the inflammatory microenvironment (by synergising with TAM/MDSC polarisation) and the development of chemotherapy tolerance (preclinical evidence supports its role in promoting docetaxel resistance) (Matsushita et al., 2022; Shan et al., 2025). In mouse models, LPS further amplifies these effects via the NF-κB–IL-6–STAT3 cascade, establishing a positive feedback loop between inflammation and proliferation that drives sustained tumour progression (Zhong et al., 2022) (Figure 2).

Additionally, gut flora regulates the enterohepatic circulation of androgens and influences systemic androgen levels. Gut bacteria can also produce androgens from glucocorticoids, which play a key role in PCa development and progression (Cross et al., 2018). Androgens are essential drivers of PCa tumorigenesis, promoting the growth and survival of PCa cells by activating the androgen receptor (AR) signaling pathway (Fujita and Nonomura, 2019). Further, intestinal flora may promote prostate cancer resistance to androgen deprivation therapy (ADT) by maintaining or increasing androgen levels (Pernigoni et al., 2021).

In summary, microbiome influence PCa in many ways, including direct action on prostate tissue, modulation of immune responses, and influencing hormone levels through metabolites. These findings support the existence of a gut–prostate axis through which intestinal microbiome may influence PCa development and response to therapy. Further understanding this axis can pave the way for microbiome-targeted strategies to prevent and treat PCa.

### 3.3 Potential role of microbiota in the treatment of urologic cancers

Traditional treatments for urologic cancers, such as surgical resection, radiotherapy, and chemotherapy remain the clinical mainstays. Among microbe-based therapies, the intravesical administration of BacillusCalmette–Guérin (BCG) is the most established and widely adopted immunotherapy for NMIBC. BCG is an attenuated live strain of *Mycobacterium bovis* that elicits a local immune response in the bladder, promoting antitumor effects through the recruitment and activation of innate and adaptive immune cells (Larsen et al., 2020). In contrast, emerging microbial products, such as Oportuzumab Monatox (OM), a recombinant fusion protein expressed in *E. coli* targeting EpCAM—, are still under clinical investigation and have not yet entered the standard of care. Although promising, these agents currently serve more as exploratory examples of microbiota-derived therapeutics than as definitive clinical options (Ungaro et al., 2022). The BCx multi-omics strategy proposed by recent research offers a viable pathway: integrating miRNA, mRNA, lncRNA, and protein as multi-omics biomarkers for monitoring early bladder cancer recurrence. In treatment, multi-omics integration not only enables visualising the tumour, but also identifying treatable vulnerabilities. For instance, systematic integration of transcriptomics, mutational profiles, copy number variations, and clinical treatment histories enables therapeutic response prediction and effective pathway targeting (Liyanage and Liyanage, 2024).

Immunotherapy, a pioneering therapy, has become a new hotspot in cancer treatment for patients with advanced malignant tumors, low survival rate, and poor prognosis (Jin et al., 2023; Bao et al., 2024). Studies have shown that the microbiome can either induce or inhibit immune checkpoint inhibitors (ICIs) to achieve the effect of combining ICIs and driving immunotherapy (Zhang S. L. et al., 2023; Zhang et al., 2024). For instance, PD-1/PD-L1 immune checkpoint inhibitors enhance the antitumor effects of T-cells by targeting tumor cell-mediated immune system suppression (Rezasoltani et al., 2021). A study revealed that mice harboring a higher relative abundance of Ruminococcaceae and Bifidobacteria in their native gut flora demonstrated enhanced antitumor immunotherapy response and improved efficacy to anti-PD-1/PD-L1 inhibitors than those with lower microbial abundance (Yousefi et al., 2024; Narote et al., 2025). Another study found that the success rate of anti-PD-1/PD-L1 therapy in patients with renal cell carcinoma (RCC) or uroepithelial carcinoma (UC) was positively correlated with the diversity of the patient’s gut microbiota (Routy et al., 2018). Therefore, targeting the microbiome for enhancing the efficacy of immunotherapy deserves more research investment.

Recent studies have highlighted the significant role of the microbiota in influencing the efficacy of neoadjuvant immunotherapy for muscle-invasive bladder cancer (MIBC). One of the studies included 42 patients with muscle-invasive bladder cancer (MIBC) who participated in the PURE-01 trial and received pembrolizumab neoadjuvant therapy administered in a 21-day cycle. Fecal samples were collected from patients prior to each treatment. The microbial community structure in the fecal samples was analysed using 16S rRNA sequencing data, and significantly different microbial communities were identified using LEfSe. This study also utilised a mouse orthotopic MB49-Luc bladder cancer model to validate the functional role of specific microbial species in ICI therapy. The study results demonstrated that distinct microbiome profiles correlated with varying responses to immunotherapy. Patients who showed favorable treatment responses frequently had enriched populations of beneficial microbes, such as the genus Sutterella, whereas nonresponsive patients showed high levels of *Ruminococcus bromii*, which is associated with poorer event-free survival (Pederzoli et al., 2024). It is hypothesized that Sutterella may enhance the efficacy of immunotherapy through local immune modulation, such as the degradation of mucosal IgA (Moon et al., 2015). Conversely, the detrimental role of *Ruminococcus bromii* observed in this context aligns with observations from other cancer immunotherapy studies, suggesting its potential role in inhibiting antitumor immune responses. These findings highlight the significant influence of the urinary (via the gut) microbiome on therapeutic outcomes during neoadjuvant immunotherapy, suggesting new avenues for optimizing treatment efficacy through microbiome modulation.

Moreover, studies show that concurrent antibiotic therapy during neoadjuvant pembrolizumab treatment, particularly with fluoroquinolones, is associated with lowered rates of complete pathological responses and recurrence-free survival. In this analysis of the prospective, Phase II, neoadjuvant immunotherapy trial (PURE-01), concomitant antibiotic use during immunotherapy was associated with lower complete response rates (15% versus 50%, hazard ratio 0.18) and poorer 24-month recurrence-free survival (63% versus 90%, hazard ratio 2.64). Among these, fluoroquinolones were associated with the most unfavourable outcomes (hazard ratio, 3.28). These results emphasize that antibiotic-induced dysbiosis disrupts the balance of the gut microbiota, thereby significantly impairing the efficacy of immunotherapy. Consequently, prudent antibiotic stewardship and targeted microbiota modulation are critical strategies for enhancing the efficacy of immunotherapy in MIBC patients (Pederzoli et al., 2021).

Additionally, probiotic-assisted therapy is also considered a means to enhance the effectiveness of cancer treatment. Oral administration of probiotic complexes containing Bifidobacterium, *Lactobacillus*, *Enterococcus*, or direct intravesical administration of probiotics, *Finegoldia*, and *Prevotella* are used to synergistically maintain the acidic environment of the urinary tract through lactic acid production, thereby inhibiting the colonization and growth of pathogenic bacteria (Russo et al., 2024; O’Callaghan and O’Toole, 2013). These probiotics may also enhance urinary immune defenses through immunomodulation, potentially reducing the incidence of bladder cancer. However, most findings remain preliminary, and their safety and efficacy must be confirmed in rigorously designed clinical trials—preferably randomized, adequately powered, and with standardized endpoints.

Future research on fecal microbial transplantation (FMT) for treating urologic cancers may become a new avenue of therapy. FMT, a therapy that modulates the gut microbiota, is expected to enhance the immunotherapeutic effect and improve the clinical prognosis of patients by restoring a healthy microbiota (Yadegar et al., 2024). Using FMT in urologic cancers, such as bladder and prostate cancersto restore the host microbial community can increase the abundance of beneficial bacteria, ameliorating the host urologic microbiota dysbiosis. However, the safety and long-term effects of FMT still need to be further validated, given that its efficacy may also be related to individual differences in patient cancer types, treatment regimens, and individual microbiota. There is still a long way to go for FMT to become a novel and established strategy for adjuvant cancer therapy.

### 3.4 Summary and outlook

Exploring the link between urologic cancers and the urinary tract microbiome is an ongoingprocess. Genomics and sequencing technologies have confirmed microbiome in the urinary tract and analyzed its distribution and features, the tip of the iceberg in microbiome research. Many clinical trials and hypotheses, such as the “gut-prostate axis,” have revealed the potential of the urogenital microbiome as oncogenic drivers, and as a biomarker for definitive diagnosis and prognostic monitoring. However, the current research on the urinary microbiome is not comprehensive as most studies have focused on the bacterial flora of the urinary tract, and the role of fungal flora in urinary tract cancers is not known. Recent studies have unveiled the potential roles of fungi (mycobiome) in tumors that can significantly influence health and diseases, although fungi represent a relatively small fraction compared to bacteria. Complex interactions between fungi, host immune systems, and other commensal microbial communities can potentially impact tumor biology through local and systemic pathways (Saftien et al., 2023). For example, Bukavina et al. (2022) provided the first evidence of significant differences in the gut fungal communities between bladder cancer patients and healthy individuals. Their findings showed that bladder cancer patients exhibited an increased abundance of *Hypocreales*, *Tremellales*, and *Dothideales*. Conversely, genera such as *Guignardia*, *Sebacina*, and *Stylonectria* were uniquely abundant in healthy controls. Significant differences were also observed in both alpha and beta diversities between bladder cancer patients and healthy individuals. Importantly, these alterations in fungal composition might influence the responses of patients to neoadjuvant chemotherapy. Specifically, patients who showed complete responses to neoadjuvant chemotherapy had significantly higher gut fungal diversity, characterized by higher *Hypocreales* abundance and reduced *Saccharomycetales* abundance. Conversely, non-responders showed an abundancce of *Saccharomycetes* and *Agaricomycetes* (Stone, 2022). Several mechanisms can be hypothesized for how fungi may modulate. For instance, studies have revealed that antifungal immune mechanisms mediated by C-type lectin receptors (CLRs) play a key role in bladder cancer development. The research team systematically assessed the expression profiles and functional characteristics of CLRs in the bladder tumor microenvironment and found that CLRs recognize fungal microbiome-related molecular patterns and remodel the tumor microenvironment by regulating immune cell infiltration. Notably, CLRs have significant potential value in immunotherapy for uroepithelial bladder cancer, and their feasibility as novel therapeutic targets is worth exploring in depth (Li T. et al., 2022). A research-oriented review by Liu et al. proposed that new cancer research phases is closely linked to fungi by dividing fungal-driven cancer research into causality-oriented intra-tumoral and prediction-oriented intestinal fungal aspects. The aim was comprehensively assess the causal relationship between fungal flora and malignant tumors from the perspective of ontogeny. The role of fungi in the tumor microenvironment has been emphasized through the induction of tumors in other sites by fungi following intestinal flora dysbiosis (Liu et al., 2024a; Zhu et al., 2021). These findings suggest an urgent need for a more in-depth exploration of the potential role and unique functions of fungal communities in urinary tract carcinogenesis and progression.

Within the field of urological tumours, existing evidence more strongly supports the concept of microbial community-level imbalances, rather than the pathogenic role of individual bacteria. A significant knowledge gap exists regarding the interaction mechanisms between urologic malignancies and the microbiome. Although studies have demonstrated the correlation between dysbiosis and tumorigenesis, there is still a lack of conclusive evidence as to whether this association is a driver or a secondary phenomenon of cancer progression. A multidimensional research strategy integrating prospective longitudinal cohort analysis and experimental model validation is needed to systematically analyze the causal biological mechanisms of the microbiome dynamics in urinary tract tumorigenesis. For instance, employing a prospective, longitudinal design with urine–tissue paired sampling at key junctures, such as diagnosis, peri-treatment, and recurrence, is of significant importance for investigating the relationship between microbiome dynamics and clinical events. Concurrently, interventional studies may be conducted, including dietary fiber modulation, targeted probiotic therapy, and microbiome preservation protocols. These aim to determine whether such interventions can alter pathway markers and improve clinical outcomes. Future breakthroughs in this field can substantially advance the translational validation of the microbiome as an early warning biomarker for tumors and provide theoretical support for developing early tumor prevention and treatment strategies by regulating the microbiome.

Advances in omics technology mean that integrated studies combining genomics, metabolomics, proteomics and other multi-omics tools can now provide a more comprehensive understanding of microbe-host interactions. These technologies will enable us to explore in depth how the microbiome affects the host’s physiology through its metabolites, gene expression and protein function, revealing its potential role in tumour formation.

## Glossary

CoNS: coagulase-negative staphylococci
RC: radical cystectomy
NMIBC: non-muscle invasive bladder cancer
PAMPs: pathogen-associated molecular patterns
PRRs: pattern recognition receptors
TLR: Toll-like receptor
JAK: Janus kinase
STAT3: signal transducer and activator of transcription 3
NF-κB: Nuclear factor kappa B
PI3K: phosphoinositide 3 kinase
PCa: prostate cancer
SCFA: short-chain fatty acid
GPCRs: G protein-coupled receptors
LPS: lipopolysaccharide
ADT: androgen deprivation therapy
BCG: BacillusCalmette–Guérin
OM: Oportuzumab Monatox
RCC: renal cell carcinoma
UC: uroepithelial carcinoma
FMT: fecal microbial transplantation
CLRs: C-type lectin receptors
